# Evolutionary analyses of mitochondrial carrier family of dictyostelids

**DOI:** 10.1186/s40064-016-3146-9

**Published:** 2016-08-31

**Authors:** Ming Gong, Qiuming Zhu, Qi Tan

**Affiliations:** 1 National Engineering Research Center of Edible Fungi; Key Laboratory of Edible Fungi Resources and Utilization (South), Ministry of Agriculture; Institute of Edible Fungi, Shanghai Academy of Agricultural Sciences, Shanghai, 201403 People’s Republic of China; 2 Department of Computer Science, University of Nebraska at Omaha, Omaha, NE 68182 USA

**Keywords:** Dictyostelids, Mitochondrial carriers, Metabolic activity, Solute transport, ADP/ATP

## Abstract

The transportation of solutes across the inner membrane of the mitochondria is catalyzed by a nuclear-coded family of transport proteins called mitochondrial carriers (MCs). Sequences from dictyostelid genome projects have facilitated analysis of the evolution of the dictyostelid mitochondrial carrier family (MCF). The average evolutionary distances between various regions in the MCF shows that the transmembrane region (TR) and conical pit region (CPR) are the only two conserved structural regions. A phylogenetic tree built using the concatenated orthologous TR and CPR sequences of 7 MCs showed that dictyostelids are similar to metazoans in this way. A close evolutionary relationship was observed between dictyostelids and metazoans in 4 MCs known to be related to ADP/ATP transport (MAA). This was further evidenced by the fact that dictyostelids have undergone gene expansion similar to that of metazoans during the evolution of MAA. Sequence logo analysis of CPR in MAA showed that dictyostelids have motifs similar to those of Metazoa. Combined with the conserved substrate binding site of 7 MCs in eukaryotes, it is postulated that dictyostelids are closely related to Metazoa with respect to the evolution of MAA.

**Electronic supplementary material:**

The online version of this article (doi:10.1186/s40064-016-3146-9) contains supplementary material, which is available to authorized users.

## Background

Social Amoebae, or Dictyostelia, are eukaryotic microbes with a unique life cycle consisting of both uni- and multicellular stages (Basu et al. 2013). Each organism starts its life as a unicellular amoeba, but when starved it aggregates with others to form a multicellular fruiting body. This process has been best described in the model organism *Dictyostelium discoideum* (Schaap et al. 2006; Romeralo et al. 2011). The phylogeny of *D. discoideum* constructed on a proteome scale indicated divergence along the branch diverged from the metazoan–fungal lineage (Eichinger et al. 2005), which means that the phylogenetic status of *D. discoideum* is distinct from that of Metazoa as the whole. However, *D. discoideum* has achieved multicellularity using strategies similar to those of Metazoa, indicating their evolutionary relationship (Eichinger et al. 2005).

Previous studies have shown the evolutionary correlation of *D. discoideum* and Metazoa in signaling systems. The *D. discoideum* genome encodes numerous G-protein-coupled frizzled/smooth receptors, metabotropic glutamate, SH2 domain based phosphoprotein signaling, and secretin families that were previously thought to be specific to animals (Sucgang et al. 2011). Molecular channels facilitate cell–cell translocation of solutes and signaling molecules and are widely distributed in almost all complex multicellular organisms (Knoll 2011). It is necessary to further study the evolutionary relationship between Dictyostelia and Metazoa in solute channel proteins.

Mitochondrial carriers (MCs) are widespread in eukaryotes and are important solute transport channels that provide a link between metabolic reactions occurring in the cytosol and those that occur in the mitochondrial matrix by catalyzing the translocation of numerous solutes across the membrane (Palmieri 2013; Palmieri and Pierri 2010; Kunji and Robinson 2010; Arco and Satrústegui 2005; Palmieri 2004). MCs are involved in many important metabolic pathways, such as oxidative phosphorylation, the citric acid cycle, fatty acid oxidation, amino acid degradation, and Ca^2+^ cell signaling (Palmieri 2004; Arco and Satrústegui 2005; Palmieri 2013). MCs have several sequence features in common: a tripartite structure, a threefold repeated signature motif, and 6 transmembrane α-helices. These structural features are different from those of any other solute carrier family and allow to unequivocally recognize MCs (Palmieri 2013). Together with the fact that MCs are the largest of solute carriers and widespread in eukaryotes (Palmieri 2013), the metabolic importance and specific structural features of MCs give them a competitive advantage in the study of the evolutionary relationship between Dictyostelia and Metazoa in solute channel proteins. Dictyostelid genome projects, which were performed on the 4 representative social amoeba dictyostelids genomes (Heidel et al. 2011; Sucgang et al. 2011), have facilitated analysis of the evolution of the mitochondrial carrier family (MCF) in Dictyostelia.

## Methods

### Detection of conserved structural region

The structure of the MCF consists of three segments: NCLOOP24, which faces the cytosol; the transmembrane region (TR), which is located in the inner membrane; and LOOP135, which faces the matrix. The conical pit region (CPR) in LOOP135 is composed of three groups with the PX[D/E]XX[K/R]X[K/R] motif and five amino acids downstream near the C-terminus. The classification of different structural region has been reported previously (Gong et al. 2010).

Average evolutionary distance (AED) was used to estimate the degree of conservation of each structural region: the smaller the mean value during evolution, the higher the conservation. To characterize the conservation of different structural regions, the AED of the corresponding structural region sequence from each orthologous subfamily of human MCF (SLC25) was calculated using MEGA 5.0 (model = Dayhoff matrix; gamma parameter = 2.0). The details of the processes by which each orthologous subfamily were determined can be found in a previous work (Gong et al. 2010).

### Phylogenetic analysis

Eukaryotic orthologous sequences of SLC25 were obtained from the InParanoid eukaryotic ortholog database (Ostlund et al. 2010). SLC25 served as text query in the search of human sequences in the InParanoid 8 database to obtain the eukaryotic orthologous sequences of SLC25. The sequences supported by the best bootstrap values in InParanoid database were selected as the orthologous sequences. Members of SLC25 with a high coverage of taxa (>90 %) in the inParanoid database were selected for phylogenetic analysis. The tandem concatenated sequences consisting of the orthologous TR and CPR sequences in these eukaryotic MCs were used to construct a phylogenetic tree. The accession numbers of these orthologous sequences can be found in Additional file 2: Table S1.

Protein sequences were aligned using Muscle with default parameters (Edgar 2004). Maximum likelihood trees were inferred using PhyML v3.0 (Guindon et al. 2010) with the LG model (Le and Gascuel 2008). Clade support was calculated using SH-like approximate likelihood ratio tests (aLRT) (Anisimova et al. 2011). PhyML analyses were performed using NNI tree topology searches with estimated Gamma shape parameters.

### Gene family size analysis of MAA

The protein sequences of 18 representative eukaryotic species were used for the comparative genomic analysis of MCs. An *E*-value of 10^−5^ was used for the BLASTp search to obtain the homologous sequences of SLC25A4, 5, 6, and 24 in the genomes of 18 representative eukaryotic species. Multigene families were generated from all of the obtained MC proteins of selected genomes using SCPS tools at default settings (BLASTp, cut-off *e*-value ≤10^−35^) (Paccanaro et al. 2006). The multigene families were then analyzed for evolutionary changes in gene family size using the CAFÉ program with the input phylogenetic tree (De Bie et al. 2006).

Because alpha tubulin provides materials for the phylogenetic analysis of species, alpha tubulin protein sequences were used to building the input phylogenetic tree for analysis of the size of the MAA gene family (Parfrey et al. 2010). The orthologous sequences of human alpha tubulin in 18 representative eukaryotic species were obtained from the InParanoid 8 database. Protein sequences were aligned using Muscle with default parameters (Edgar 2004). Maximum likelihood trees were produced using PhyML v3.0, with the LG model (Guindon et al. 2010; Le and Gascuel 2008).

## Results and discussion

### Conserved structural regions in MCF

Although MCs display a wide variety of transported solutes (i.e., a large variety of metabolites, nucleotides and coenzymes), all MCs have common sequence features: a tripartite structure, a threefold repeated signature motif PX[D/E]XX[K/R]X[K/R], and six transmembrane a-helices (Palmieri 2013). These common structural features allow the program to treat MCs as a single unit for study of the evolution of MCF.

**Fig. 1:**
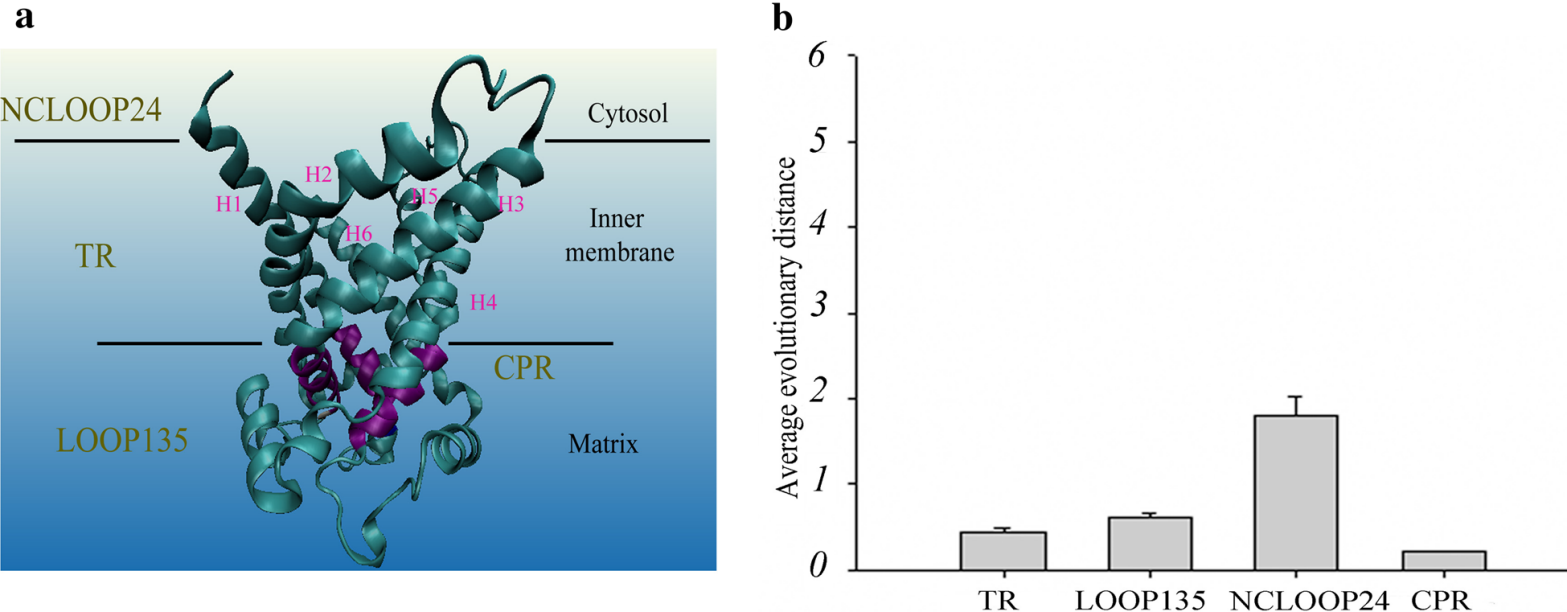
The conserved regions of MCs. **a** The three-dimensional structure of the ADP/ATP carrier in *Bos taurus* (PDB code: 1OKC). The region marked in purple represents the CPR. The protein structure was drawn using VMD (Humphrey et al. 1996). **b** Average evolutionary distances of different regions in the MCF

The 3D structure of ADP/ATP has been used as a template for the study of various MCs (Palmieri 2013; Pebay-Peyroula et al. 2003). For this reason, the spatial distribution of TR and CPR was here depicted using the 3D structure of the ADP/ATP (PDB code: 1OKC). A six-transmembrane a-helix bundle (H1–H6) consisted of TR (Fig. 1a). The CPR 1, 3, and 5 consisting of the threefold repeated signature motifs PX[D/E]XX[K/R]X[K/R] and their C-terminal 13 sites are located at the C-terminus of the three odd-numbered transmembrane α-helices, respectively (Fig. 1a). The average evolutionary distances of various regions in the MCF showed that the TR and CPR were the only two regions conserved during evolution (Fig. 1b). Combined with the TR and CPR, which constitute the core of solute carrier transport (Gong et al. 2010), these observations indicated that they can represent MCF.

### Evolutionary similarity of dictyostelids and metazoans with respect to MCs

**Fig. 2:**
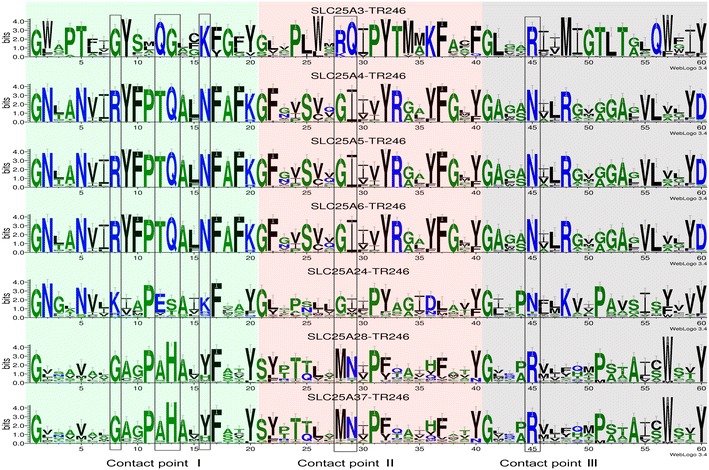
Logo analysis of TR246 in the orthologous sequences of 7 SLC25s in eukaryotes. *Different color shading* from left to right indicates TR2, 4, and 6, respectively. The orthologous sequences of 7 SLC25s from 48 eukaryotic taxa in Additional file 2: Table S3 were selected for logo analysis of TR246. Contact point I, II, and III indicate substrate contact sites. WebLogo 3 was used for the logo analysis (Crooks et al. 2004)

The distribution of orthologous sequences of human MCs (SLC25) showed SLC25A3, 4, 5, 6, 24, 28, and 37 to have the most coverage (>90 %) in the Inparanoid eukaryotic ortholog database (Additional file 2: Table S2), indicating the orthologous sequences of 7 SLC25s that were found to be widely distributed in eukaryotes. Detailed information regarding these 7 SLC25s is given in Table 1. These 7 SLC25s and their orthologous sequences (7 MCs) were to represent the MCF. Because the common substrate binding site has already been identified in the three even α-helices (TR246) of MCs, attention was shifted to variation in substrates transported by these 7 MCs in eukaryotic evolution (Robinson and Kunji 2006). The TR246 sequences were concatenated according to the order of the even transmembrane α-helices (H2, 4, and 6) from the N-terminal to C-terminal (Fig. 1a). Logo analysis of the concatenated TR246 showed that the residues in substrate binding site of 7 MCs were basically conserved in eukaryotic evolution (Fig. 2), which provided evidence for the similar types of substrates transported by 7 MCs in eukaryotic evolution. Together with many conserved signature motifs in the CPR of 7 MCs (Table 1), these observations indicate that the phylogenetic tree constructed using the concatenated TR and CPR of 7 MCs may reflect solute transport capabilities.

**Table 1:**
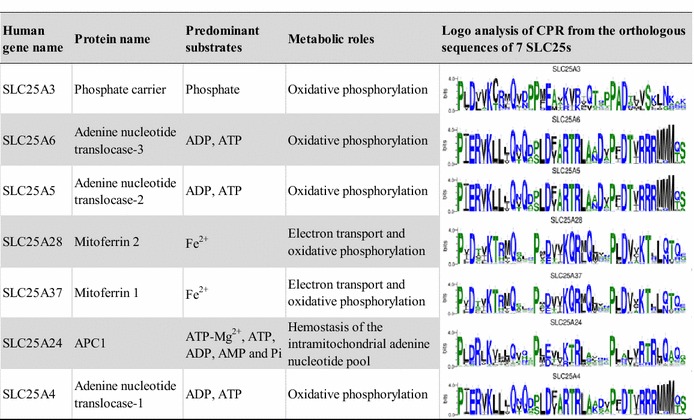
7 SLC25s widely distributed in eukaryotes

7 SLC25s were ranked by coverage score. High coverage score (≥90 %) of 7 SLC25s in 230 eukaryotic taxa of InParanoid database can be found in Additional file 2: Table S2. The concatenated CPR1, 3, and 5 of orthologous sequences of these 7 SLC25s in 48 taxa were used for the logo analysis. Detailed information regarding SLC25 can be found in reference (Palmieri 2013)

**Fig. 3:**
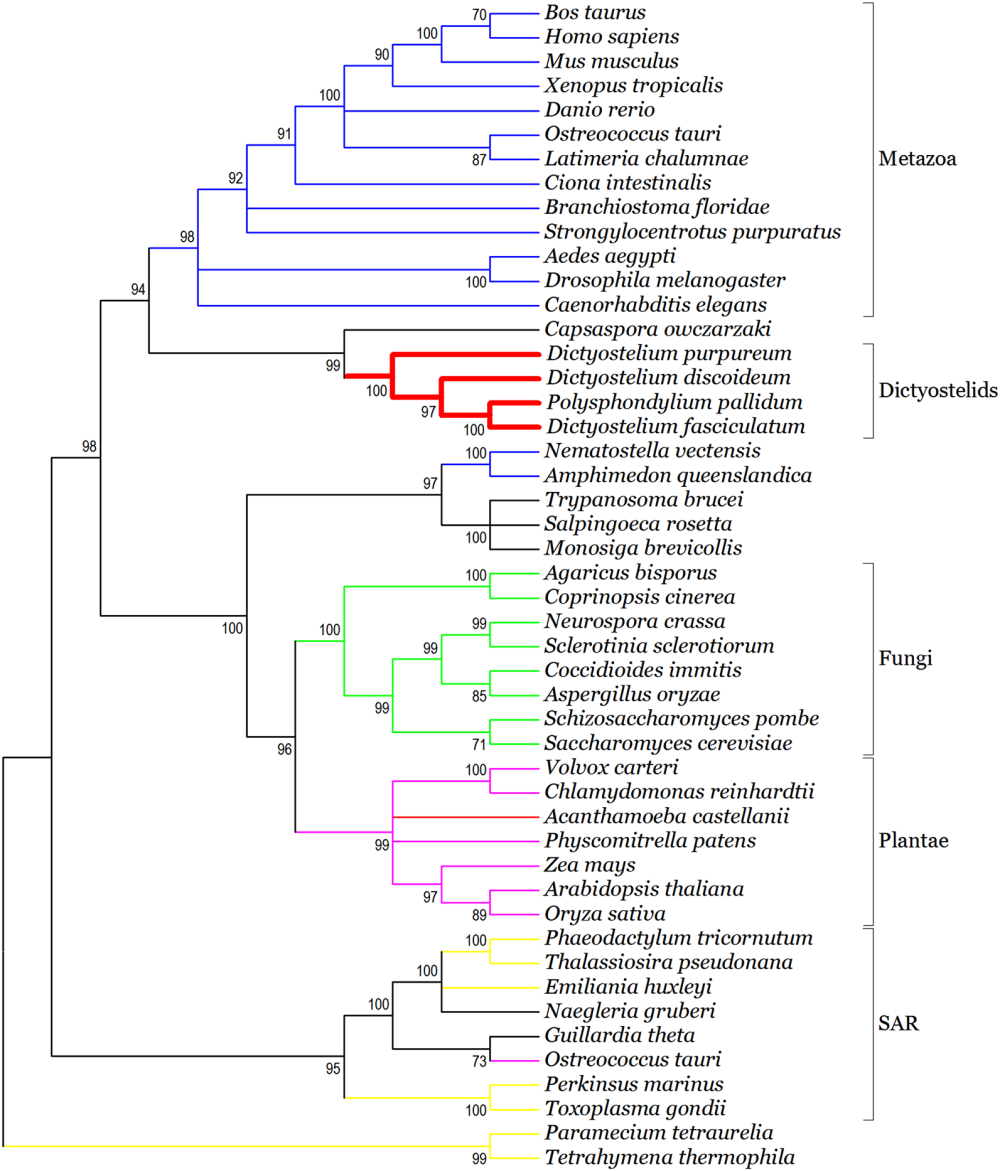
Maximum likelihood tree of eukaryotes reconstructed with the tandem concatenated orthologous TR and CPR sequences of 7 SLC25s in 48 taxa. Branch support was given as SH-like aLRT scores. Only SH-like aLRT scores above 70 % are shown. *Bold red* branches represent the dictyostelid cluster. SAR is a clade that includes stramenopiles, alveolates, and Rhizaria. The first letter of each group provides the “SAR” in the name. The details regarding the distribution of orthologous sequences of SLC25 in InParanoid database can be found in Additional file 2: Table S3

The TR and CPR sequences were concatenated according to the order of the 6 transmembrane α-helices (H1–H6) and CPR (CPR1, 3, and 5) from the N-terminal to C-terminal (Fig. 1a). Here, 7 MCs were concatenated in the order of the SLC25 classification number, from low to high (SLC25A3, 4, 5, 6, 24, 28, and 37). The tree constructed using the concatenated orthologous TR and CPR of 7 MCs with the average sequence length (1040) clearly showed five clusters, the Metazoa, dictyostelids, fungi, plantae, and SAR (Fig. 3). Phylogenetic tree showed that four dictyostelids were clustered close to Metazoa (Fig. 3). These observations indicate dictyostelids may have solute transport mechanisms similar to those of Metazoa. This is partially consistent with the fact that *D. discoideum* amoebae display distinct characteristics of true multicellularity, such as cell–cell signaling, cellular specialization, and coherent cell movement (Raper 1984; Kessin 2001). As the only solitary free-living representative Amoebozoa, *Acanthamoeba castellanii* has been used a reference for studying the evolution of dictyostelid MCF (Clarke et al. 2013). In contrast, the Amoebozoa *A. castellanii* was not found near Metazoa or the dictyostelid cluster (Fig. 3). The MC-based phylogenetic tree produced here showed *Amphimedon queenslandica* and *Nematostella vectensis* belonging to basal metazoans not to be nested within any metazoan group. This suggests that they cannot transport solutes any more effectively than other complex metazoans to satisfy the energy needs of complex metabolic activities. This was evidenced by the fact that no molecular functions related to metabolism were found to be enriched in an enrichment analysis of molecular functions of basal metazoans (Srivastava et al. 2010). To some extent, these observations suggest that dictyostelids have a more developed capacity to transport solutes, which would satisfy the energy needs of true multicellularity.

### Evolutionary similarity of dictyostelids and metazoans with respect to MAA evolution

Further phylogenetic analysis using the orthologous TR and CPR sequences of 7 SLC25A showed the close evolutionary relationship of the orthologous sequences of SLC25A4-5-6 and SLC25A24 between dictyostelids and metazoans (Additional file 1: Figure S1). The 4 MCs, are mainly responsible for transporting ADP/ATP (Palmieri 2013). This indicated the close evolutionary relationship between dictyostelids and metazoans with respect to these 4 MCs, which are known to be related to ADP/ATP transport (MAA).

**Fig. 4:**
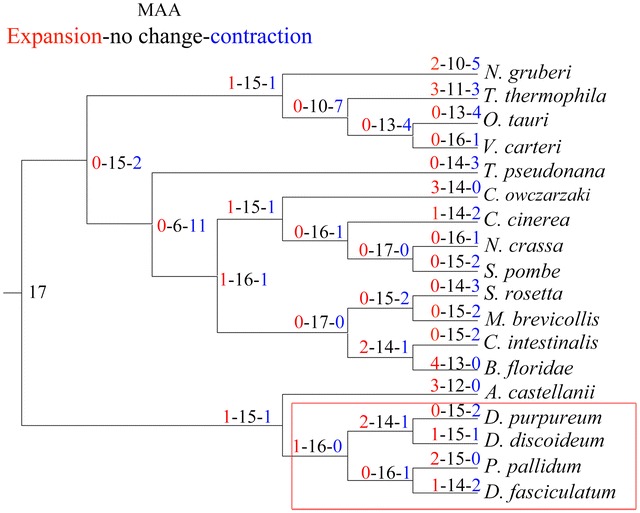
Evolutionary changes in the eukaryotic MAA. The figure represents the total number of MAA subfamilies in each species or node. The numerals on branches show the numbers of expanded (*left*, *red*), unchanged (*middle*, *black*), and contracted (*right*, *blue*) MAA subfamilies along the lineages. The *red box* contains the four dictyostelids

Attention was shifted to the evolutionary changes in subfamilies of the MAA in eukaryotes. The phylogenetic tree built using alpha tubulin protein sequences showed that fungi (*Coprinopsis cinerea* and *Neurospora crassa*) and invertebrate chordates (*Ciona intestinalis* and *Branchiostoma floridae*) consisted of a group instead of dictyostelids. This was consistent with the phylogeny of *D. discoideum* constructed on a proteome scale (Eichinger et al. 2005). Then, the phylogenetic tree using alpha tubulin protein sequences was used for the analysis of evolutionary changes in the size of the MAA gene family. However, although gene expansion has been observed in the nodes connected to the MAA of most model invertebrate chordates, gene expansion was not observed in the nodes leading to fungi MAA, only in the nodes leading to dictyostelids MAA (Fig. 4). Together with the close phylogenetic relationship between dictyostelids and metazoans in the MAA (Additional file 1: Figure S1), these observations indicated that dictyostelids were closely related to Metazoa in the evolution of MAA.

### Common motifs shared by dictyostelid and metazoan MAA

After the phylogenetic analysis of MCs in Fig. 3, sequence logo analysis of the most conserved CPR region (Fig. 1b) was conducted in the MAA of the three groups (Dictystelids, Metazoa, and Fungi). Results showed that dictyostelids had a motif more similar to that of Metazoa rather than to that of fungi (Additional file 1: Figure S2), at such sites as 1, 11, 12, and 13. Combined with the conserved substrate binding site in the TR246 of 7 MCs (Fig. 2), these observations indicated that dictyostelids shared the common motif with Metazoa in the conserved TR and CPR of MAA.

Because the charged residues in the conserved motif of CPR formed a salt bridge network (Robinson et al. 2008; Robinson and Kunji 2006), attention was shifted to the polar residues in the CPR motif. The proportion of polar glutamine (59.3 %) in the twelfth position (Q_12_) of CPR near the C-terminus in dictyostelids is similar to that (59.6 %) of Metazoa, which is markedly higher than that of fungi (33.3 %). The polar NH_2_ group on the long-chain branch of Q was found to strengthen the salt bridge network through participation in the hydrogen bonding reaction. Because the salt bridge network in CPR is involved in substrate transport, this indicates the enriched Q_12_ in CPR may be associated closely with substrate transport (Robinson et al. 2008). The common motif shared by dictyostelids and metazoans in MAA indicate that dictyostelid MAA may be capable of ADP/ATP transport similar to that performed by Metazoa MAA.

## Conclusions

The MC-based phylogenetic tree described here showed that dictyostelids were similar to metazoans in the evolution of the MAA. Dictyostelid MAA exhibited gene expansion similar to that of metazoan MAA. Further sequence logo analysis of the conserved TR and CPR in MAA showed that dictyostelids had a motif similar to that observed in Metazoa. These findings may aid understanding of the evolutionary correlation between dictyostelids and Metazoa in MAA.

## Additional files

10.1186/s40064-016-3146-9 **Figure S1.** Maximum likelihood tree of eukaryotes reconstructed with the orthologous TR and CPR sequences of 7 SLC25. Branch support was given in the form of SH-like aLRT scores. Red branches represent the metazoans cluster. Blue branches represent the dictyostelid cluster. SLC25A4-5-6 represents the orthologous TR and CPR sequences of SLC25A4, 5, and 6 in 48 taxa. **Figure S2.** Logo analysis of CPR in the MAA. The MAA sequences from the species in the three groups based on the phylogenetic tree were used for the logo analysis (Figure 3).
10.1186/s40064-016-3146-9 **Table S1.** Accession numbers of the orthologous sequences of 7 SLC25 used in the MCs-based phylogenetic tree. **Table S2.** Orthologous sequences distribution of SLC25 in 230 taxa in the InParanoid database. High coverage of SLC25 was marked by brown shading. **Table S3.** Distribution of orthologous sequences of SLC25 in 41 taxa of InParanoid database. Orthologous sequences of 7 SLC25 (blue shading) with 0 hits (gray shading) were obtained using reciprocal best hits BLAST in NCBI non-redundant database. The orthologous sequences in 6 other taxa were obtained using reciprocal best hits BLAST in NCBI non-redundant database.
