# Supplementary material for: Evolutionary analyses of mitochondrial carrier family of dictyostelids
Source: Springerplus. 2016 Aug 31;5(1):1465. doi: 10.1186/s40064-016-3146-9 (PMC5007236; doi:10.1186/s40064-016-3146-9)
Supplement: Supplementary file 1 — 10.1186/s40064-016-3146-9 Figure S1. Maximum likelihood tree of eukaryotes reconstructed with the orthologous TR and CPR sequences of 7 SLC25. Branch support was given in the form of SH-like aLRT scores. Red branches represent the metazoans cluster. Blue branches represent the dictyostelid cluster. SLC25A4-5-6 represents the orthologous TR and CPR sequences of SLC25A4, 5, and 6 in 48 taxa. Figure S2. Logo analysis of CPR in the MAA. The MAA sequences from the species in the three groups based on the phylogenetic tree were used for the logo analysis (Figure 3). [file 40064_2016_3146_MOESM1_ESM.doc]

**Supplementary material**

**Figure S1 Maximum likelihood tree of eukaryotes reconstructed with the orthologous TR and CPR sequences of 7 SLC25.** Branch support was given in the form of SH-like aLRT scores. Red branches represent the metazoans cluster. Blue branches represent the dictyostelid cluster. SLC25A4-5-6 represents the orthologous TR and CPR sequences of SLC25A4, 5, and 6 in 48 taxa.

**Figure S2** **Logo analysis of CPR in the MAA.** The MAA sequences from the species in the three groups based on the phylogenetic tree were used for the logo analysis (Figure 3).


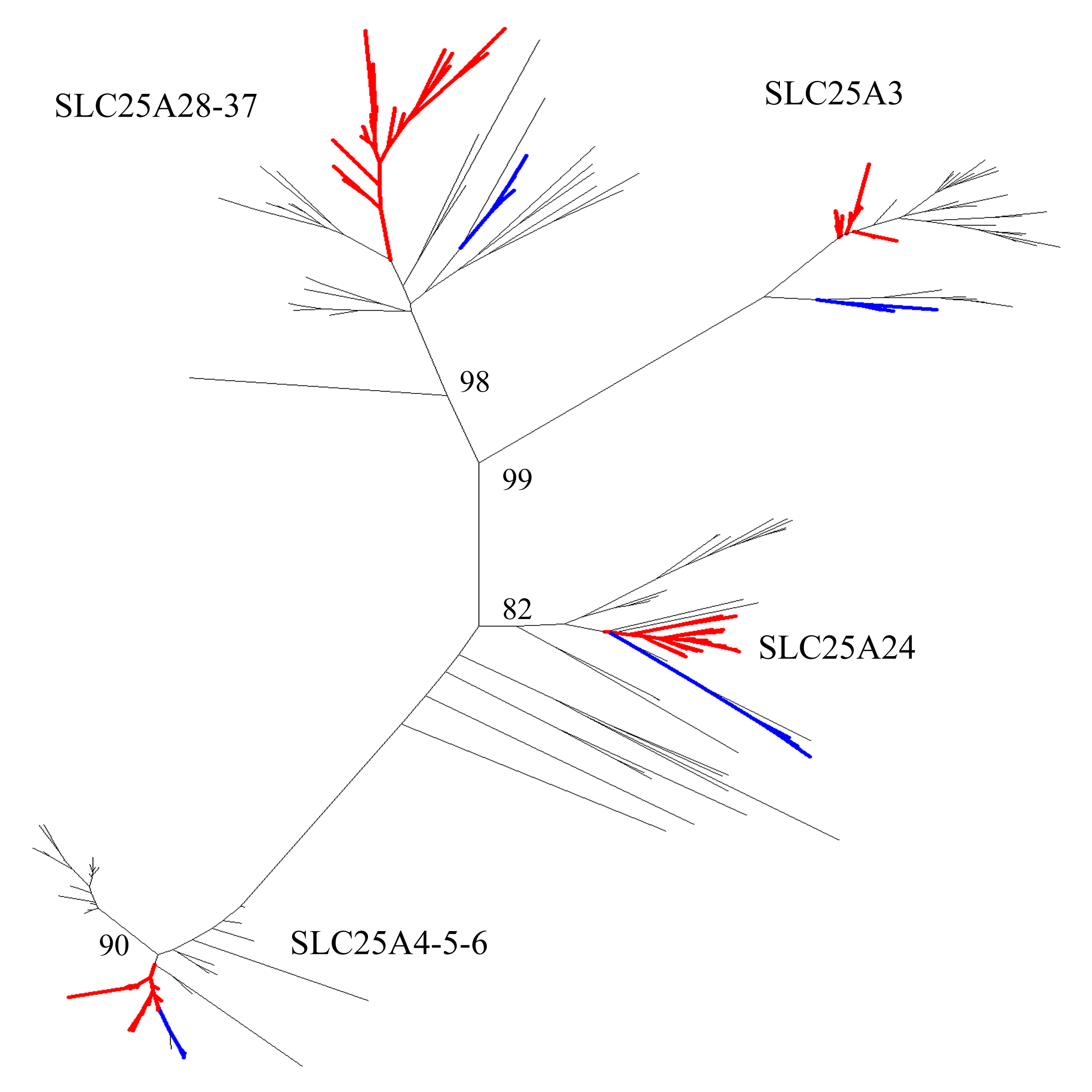


Fig. S1


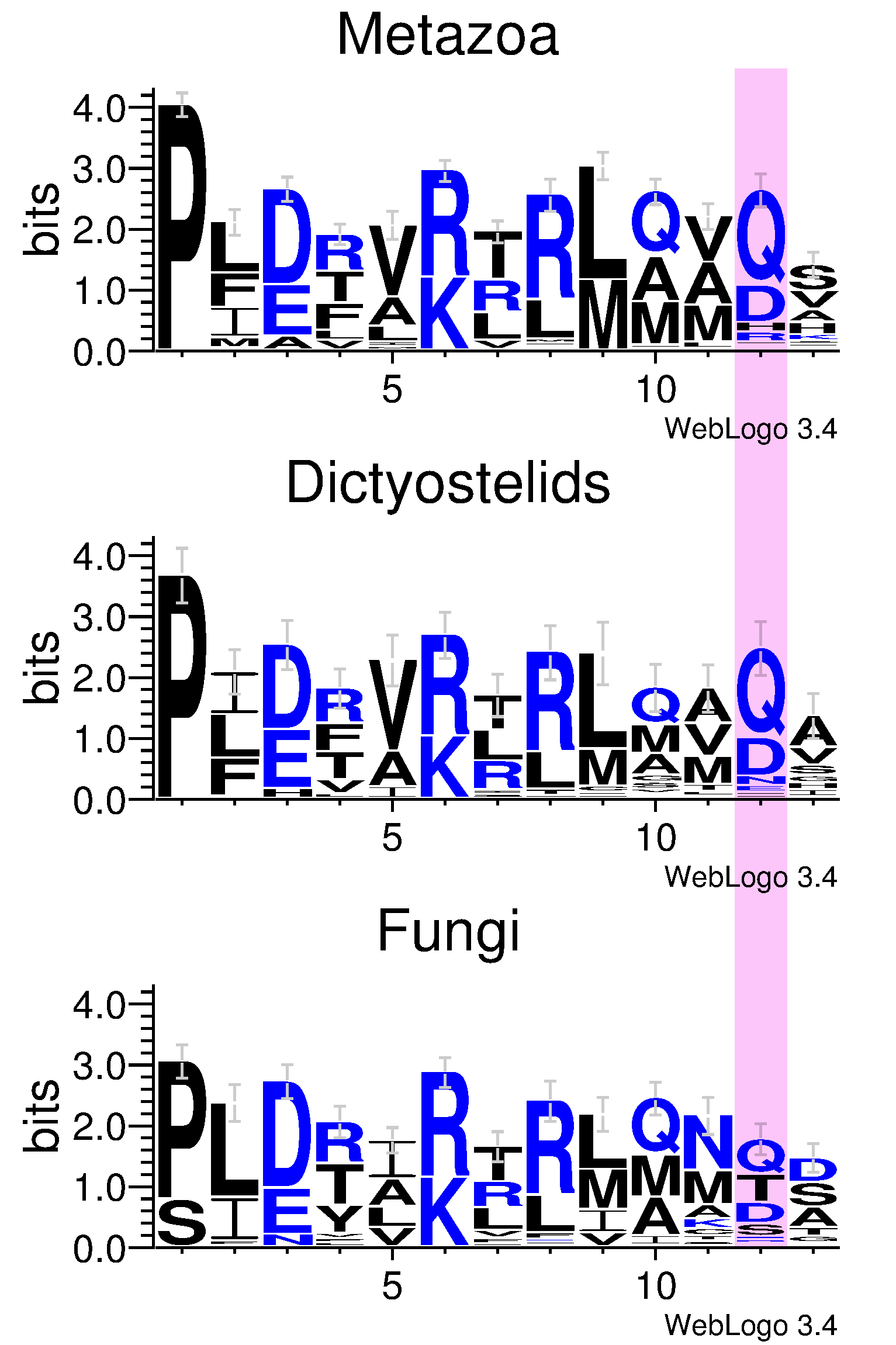


Fig. S2
